# IMPACT OF 4MS ON OUTCOMES FOR HOSPITALIZED OLDER ADULTS IN AN AGE-FRIENDLY SYSTEM

**DOI:** 10.1093/geroni/igae098.4000

**Published:** 2024-12-31

**Authors:** Justin Lam, Susan Kwiatek, Maria Carney, Philip Solomon, Mark Tursi, Christian Nouryan, Edith Burns

**Affiliations:** Zucker School of Medicine at Hofstra/Northwell, Brooklyn, New York, United States; Northwell Health, New Hyde Park, New York, United States; Zucker School of Medicine at Hofstra/Northwell, New Hyde Park, New York, United States; Zucker School of Medicine at Hofstra/Northwell, New Hyde Park, New York, United States; Krasnoff Quality Management Institute/Northwell Health, New Hyde Park, New York, United States; Zucker School of Medicine at Hofstra/Northwell, Manhasset, New York, United States; Northwell Health, Manhasset, New York, United States

## Abstract

**Introduction:**

The Institute for Healthcare Improvement identifies four essential components (the 4Ms) of high-quality care for older adults within an Age-Friendly Health System (AFHS): What Matters, Medication, Mentation, and Mobility. This framework allows healthcare providers to address and act on key issues, such as goals-of-care discussions, the in-patient use of potentially harmful medications (e.g. benzodiazepines, opioids, hypnotics), delirium prevention, and mobility maintenance.

**Methods:**

EMR data pulled manually and through aggregated dashboards. Population/Setting: patients ≥65 admitted June-July 2024 to eight hospitals (5 community, 2 tertiary, 1 quaternary) within a large integrated health system, all committed to care excellence. Patients grouped into categories based on the number of assessed M’s. Positive screens were reviewed for “act-ons.” Patient outcomes: LOS, disposition, 30-day readmissions and ED visits.

**Results:**

Of 2,337 adults aged ≥65, 0.3% had no assessments, 10.6% had one assessment, 39.6% two assessments, 40.3% three assessments, and 9.2% had all four assessments. Of the 118 patients who had all 4Ms assessed, chart review showed 42% had an ‘act-on’ for What Matters, 43% act-on for Medication, 22% act-on for Mentation, 83% act-on for Mobility. Mean LOS 11.8 days (range: 3-72); June 30-day readmissions: 10.2%. Conclusions: A minority of patients had all 4Ms assessed. Act-ons were documented most frequently for Mobility, less for other M’s. Data on outcomes for ≤3M assessments is being analyzed and compared to those with 4Ms. This data will contribute to the growing evidence base for all 4Ms in AFHS transformation.

